# Two decades of loneliness among children and adolescents: longitudinal trends, risks and resources – Results from the German BELLA and COPSY studies

**DOI:** 10.1007/s00787-025-02779-6

**Published:** 2025-06-09

**Authors:** F. Zoellner, M. Erhart, R. Schütz, A.-K. Napp, J. Devine, F. Reiss, Ulrike Ravens-Sieberer, Anne Kaman

**Affiliations:** 1 Department of Child and Adolescent Psychiatry, Psychotherapy and Psychosomatics, University Medical Center Hamburg Eppendorf Center for Psychosocial Medicine, Research Division “Child Public Health”, Martinistrasse 52, W 29, 20246 Hamburg, Germany; 2 Alice Salomon University of Applied Sciences, Berlin, Germany; 3 Brandenburg University of Technology Cottbus-Senftenberg, Cottbus, Germany

**Keywords:** Loneliness, Mental health, Risk factors, Youth, Longitudinal, COVID-19

## Abstract

Loneliness among young people is a significant public health concern, yet research on its prevalence, longitudinal trends, and associated risk and resource factors in Germany remains limited. This paper draws on two German longitudinal panel surveys with refreshment sampling – the BELLA study (2003–2017) and the subsequent COPSY study (2020–2024), and includes ten nationwide measurement points. The sample comprised *N* = 3,043 participants (49.9% female; age range 11–17 years) from the BELLA study, and *N* = 1,909 participants (51.0% female; age range 11–17 years) from the COPSY study. Our findings indicate that loneliness rates remained relatively stable between 2003 and 2017 (9.5–15.5%) but spiked dramatically in 2020 (34.6%) during the COVID-19 pandemic. By 2024, 20.5% of young people still reported feeling lonely at least sometimes during the past week. Consistently, girls were more likely to report loneliness than boys. Mixed model panel regression analyses identified key risk factors for loneliness, like female gender, screen time, parental mental illness, as well as mental and physical health complaints in children and adolescents. In contrast, personal, familial, and social resources were associated with lower levels of loneliness. While some degree of loneliness is a common experience in youth, persistent loneliness poses a risk to mental and physical health. Our findings underscore the need for targeted interventions, particularly in school, leisure, and family settings, where social and emotional skills can be fostered.

**Supplementary Information:**

The online version contains supplementary material available at 10.1007/s00787-025-02779-6.

## Introduction

The high prevalence of loneliness among young people represents a significant public health challenge. By 2023, the World Health Organization (WHO) formally recognized loneliness as a “global public health concern” [22]. Additionally, the Surgeon General, the leading US public health official, has expressed deep concerns regarding the severe impacts of what he has termed an “epidemic of loneliness and isolation” [15]. The COVID-19 pandemic exacerbated feelings of isolation through lockdowns, remote learning, and social distancing measures [12], but research indicates that the trend of increasing loneliness began as early as 2012 [50].

Most researchers agree that loneliness refers to a subjectively perceived and distressing discrepancy between desired and actual relationships [33]. Experiencing loneliness differs from the experience of being alone, which can have positive effects [1]. The perceived lack of social connections poses a significant threat to both physical and mental health and longevity [12, 27, 48]. Loneliness and social isolation have been shown to increase the risk of premature death in adults by 26% and 29%, respectively [19].

Loneliness among young people can vary in intensity, with many experiencing it as short-lived or situational. However, problematic and chronic levels of loneliness in young people are widespread. A systematic review of data from 113 countries collected between 2000 and 2019 found loneliness prevalence ranged from 9.2% in South-East Asia to 14.4% in the Eastern Mediterranean region [48] in 12 to 17 years old adolescents. This review does not report rates for European adolescents due to limited data and inconsistent measures. But in *Health Behaviour in School-aged Children* (HBSC) studies, the prevalence of loneliness in 11- to 15-year-olds ranged from 6.3% in Denmark [28] to 8.2% in the UK [35] to 15% in Finland [27]. In Germany, research on loneliness among children and adolescents is limited. A regional study in Brandenburg found loneliness rates between 10.8% and 16.8% among 11- to 15-year-olds [46].

Longitudinal studies on loneliness are relatively rare, making it difficult to track long-term trends among young people. According to the *Programme for International Student Assessment* (PISA), almost twice as many young people worldwide said they felt lonely in 2018 compared to 2000, with a steep increase after 2012 [50]. Danish adolescents experienced a rise in loneliness from 1991 to 2014 [28], while in Finland, loneliness among adolescents increased from 11 to 15% between 2006 and 2018 [27]. By contrast, the UK saw stable rates of 8.2% between 2006 and 2014 [35]. During the COVID-19 pandemic, systematic reviews of longitudinal studies identified a significant increase of loneliness in young people compared to pre-pandemic levels, although the effect size was small [11, 12]. However, a large Norwegian study on adolescents reported a steady, linear increase in loneliness from 2014 to 2021, with no significant additional rise linked to the onset of the COVID-19 pandemic, suggesting that the pandemic may have simply continued an existing trend [52]. Post-pandemic, evidence on recovery in young people remains limited. One of the few studies addressing this question, conducted in the Netherlands, found that while most adult age groups returned to pre-pandemic levels of loneliness, recovery among emerging adults (16 to 24 years) appeared incomplete [51]. In Germany, comprehensive longitudinal data on loneliness in young people are still lacking.

Several factors can contribute to feelings of loneliness, including gender, age, and various social and health-related variables. In terms of gender, numerous studies have consistently reported a higher prevalence of loneliness among girls compared to boys [9, 27, 28, 50]. Regarding age, most research shows that loneliness tends to increase with age during childhood [9, 27, 35]. However, some studies have found no significant age-related differences in loneliness among young people [28]. With regard to socioeconomic status, adolescents from families with lower socioeconomic status (SES) were found to be lonelier, i.e., there was an inverse association between SES with loneliness [28, 35]. Excessive screen time and social media use may also contribute to loneliness in children and adolescents by reducing face-to-face interactions [49]. Further, parental mental illness is a well-established risk factor for their children’s mental health [56] and is associated with increased loneliness in children and adolescents [20]. Higher levels of loneliness in young people have been linked to physical health complaints, such as headaches and stomachaches [27, 35], as well as mental health issues, including depression [34], anxiety [4], sleep problems [9, 27], and suicidality [4]. Childhood loneliness is also a specific risk factor for adult psychiatric disorders [54].

On the other hand, our previous epidemiological research found that resource factors, such as self-efficacy, positive family climate, and social support, were associated with fewer mental health symptoms [24, 56]. International findings show that strong social connections with peers and within the family are associated with less loneliness in young people [6, 55].

Longitudinal research is essential for understanding how mental health develops across childhood and adolescence, as it enables the identification of age-specific risk and protective factors, the monitoring of developmental trajectories, and the planning of timely and effective interventions. In Germany, the BELLA (2003–2017) [32] and the COPSY (2020–2024) [40] studies, both designed as nationwide longitudinal panel surveys with refreshment sampling, have examined the mental health and well-being of young people over the past two decades.

Despite these efforts, there is still a lack of systematic longitudinal data on the development and course of loneliness, and its associated risk and resource factors in Germany across an extended period. To address this gap, we analyzed data from the BELLA and COPSY studies to investigate the following research questions across three domains:

I. Current frequency of loneliness in German young people (2022–2024):

1. How prevalent is loneliness among young people?
2. Are there significant gender differences in loneliness?
3. Are there significant age differences in loneliness?

II. Trends of loneliness in German young people over the past 20 years (2003–2024):

1. How has loneliness among children and adolescents evolved from 2003 until 2017 (BELLA study)?
2. Did loneliness increase during the COVID-19 pandemic (COPSY study)?
3. If the prevalence rates have risen during the pandemic, have they returned to pre-pandemic levels in the most recent measures (COPSY study)?
4. Are there differences by gender and age?

III. Risk and resource factors of loneliness in German young people (2022–2024):

1. Are socioeconomic and parental risk factors (e.g., older age, female gender, low parental education, single-parenthood, migration background, and parental mental illness) associated with higher loneliness in children and adolescents?
2. Is more screen time associated with higher loneliness in children and adolescents?
3. Are more mental and physical health problems in children and adolescents associated with more loneliness?
4. Are resource factors (i.e., personal resources, family cohesion, social support) associated with less loneliness in children and adolescents?

## Methods

### Studies and participants

This paper is based on two German nationwide longitudinal panel surveys with refreshment sampling examining the mental health and well-being of children and adolescents: The BELLA study (2003–2017) and the COPSY study (2020–2024).

The BELLA study (Behaviour and Well-being of Children and Adolescents in Germany) served as the mental health module of the German Health Interview and Examination Survey for Children and Adolescents (KiGGS). The baseline survey (B0, 2003–2006) included 2,863 children and adolescents aged 7–17 years, with follow-ups conducted one year later (B1, 2004–2007, *n* = 2,423), two years later (B2, 2005–2008, *n* = 2,190), six years later (B3, 2009–2012, *n* = 3,840), and eleven years later (B4, 2014–2017, *n* = 3,492). Each wave of data collection spanned three years, with the first three waves overlapping timewise. Sample selection for KiGGS (*N* = 17,641) involved stratified sampling of 167 sample points across Germany. The BELLA sample is derived as a random subset of the KiGGS sample. The first four BELLA waves used paper-pencil questionnaires and computer-assisted telephone interviews, while the last wave was conducted online. The initial response rate in KiGGS was 66.6%, and 97.3% in the sub-sample invited for the BELLA baseline [38]. Further details on the study design and sampling are available elsewhere [32, 37, 38].

The COPSY study (*CO*VID-19 and *Psy*chological Health) started at the beginning of the COVID-19 pandemic (C1, May–June 2020, *n* = 1,586). Subsequent assessments were conducted in December 2020–January 2021 (C2, *n* = 1,625), September–October 2021 (C3, *n* = 1,618), February 2022 (C4, *n* = 1,668), September–October 2022 (C5, *n* = 1,701), October–November 2023 (C6, *n* = 1,673), and October 2024 (C7, *n* = 1,505). In April 2023, all remaining COVID-19 restrictions, such as mask mandates, were lifted in Germany, and in May 2023, the WHO declared the international emergency caused by the COVID pandemic to be over. We therefore considered the last two data collection points to be post-pandemic. Families were recruited via an online panel using quota sampling to reflect the sociodemographic characteristics of the German population. The initial response rate was 46.8%, with participants completing an average of 56.7% of the waves. More information on sampling and recruitment is available elsewhere [39, 40].

For participants aged 11 to 18, data were collected via self-report, and for children and adolescents aged 7 to 18, via parent-report in each study. In both studies, families who had previously participated were re-invited for follow-ups, and new participants were recruited to counteract attrition and ensure representativeness. Data from the studies were weighted to reflect the sociodemographic characteristics of the German population [32, 41]. Across the two studies, equivalent, validated, and internationally established instruments were used to assess loneliness, mental and physical health, and resource factors in children and adolescents.

For the current analyses, we focused on participants aged 11 to 17 years, as loneliness was assessed via self-report, which was only administered to individuals aged 11 and older. Individuals with missing data in some variables were included in those analyses, if sufficient data were available. We included data from all five waves from the BELLA study, resulting in a total sample of 3,043 participants (49.9% female; age range 11–17 years, mean age = 14.2; SD = 1.8). For the COPSY study, we included one assessment per year, resulting in five time points (C1, C3, C5, C6, and C7). The sample of this study consists of 1,909 participants (51.0% female; age range 11–17 years, mean age = 14.2; SD = 3.0). Detailed information on the subsamples can be found in Table 1.

**Table 1:** Sociodemographic characteristics

	2003–2006 B0	2009–2012 B3	2014–2017 B4	2020 C1	2021 C3	2022-24 C5-C7^a^
	*n* = 1734	*n* = 1282	*n* = 1109	*n* = 1040	*n* = 1043	*n* = 1190
**Age [M (SD)]**	13.9 (2.0)	14.5 (1.8)	13.85 (1.9)	14.33 (1.9)	14.4 (2.2)	14.10 (2.7)^b^
11–13 years [n (%)]	778 (44.9)	378 (29.5)	514 (46.3)	351 (33.8)	373 (35.8)	532 (44.7)^b^
14–17 years [n (%)]	946 (54.6)	904 (70.5)	595 (53.7)	689 (66.2)	670 (64.2)	658 (53.3)^b^
**Gender**						
Male [n (%)]	865 (49.9)	633 (49.4)	515 (46.4)	508 48.8)	490 (47.0)	590 (49.6)
Female [n (%)]	869 (50.1)	649 (50.6)	594 (53.6)	531 (51.1)	544 (52.2)	592 (49.7)
Divers [n (%)]	n.a.	n.a.	n.a.	1 (0.1)	8 (0.8)	8 (0.7)
**Parental education**						
Low [n (%)]	288 (16.6)	102 (8.0)	67 (6.1)	192 (18.9)	180 (17.6)	212 (17.8)
Moderate [n (%)]	971 (56.5)	723 (56.5)	581 (53.0)	548 (53.9)	597 (58.3)	698 (58.7)
High [n (%)]	461 (26.8)	455 (35.5)	448 (40.9)	277 (27.2)	247 (24.1)	280 (23.5)
**Migration background**						
No [n (%)]	845 (87.0)	1130 (88.1)	969 (87.7)	879 (84.5)	852 (82.4)	963 (81.7)
Yes [n (%)]	126 (13.0)	152 (11.9)	136 (12.3)	161 (15.5)	182 (17.6)	215 (18.3)
**Single parenthood**						
No [n (%)]	1420 (81.2)	n.a.	n.a.	826 (79.4 )	837 (80.2)	963 (81.7)
Yes [n (%)]	324 (18.8)	n.a.	n.a.	214 (20.6)	206 (19.8)	215 (18.3)

Unweighted data; ^a^ Summarized waves (C5-C7) included in the panel regression; ^b^ in 2023

## Measures

### Loneliness

Loneliness was measured using a single item from the KIDSCREEN questionnaire: “Have you felt lonely?” [36]. Respondents were asked to think about the last week while answering. Response options ranged from 1= “never”, 2 = “almost never”, 3 = ”sometimes”, 4 = ”almost always”, to 5 = “always”. Single items for assessing loneliness are highly correlated with multi-item scales and are considered reliable and valid [31, 42]. There are no consensual agreed-upon cut-off values for loneliness [9]. For the one-week prevalence, we applied a cut-off of ≥ 3, meaning that participants reported feeling lonely “sometimes”, “almost always”, or “always” during the past week. While this cut-off includes those who feel lonely “sometimes”, and some level of loneliness is considered unproblematic, this cut-off is intended to be sensitive enough to identify children who may require closer attention, as they experience loneliness at least occasionally.

Additionally, we measured loneliness by implementing the UCLA Loneliness Scale [44] starting from COPSY C3 in 2022. Children and adolescents rated four statements regarding how they felt over the past 12 months (i.e., “I feel isolated from others”, “I feel left out”, “I lack companionship”, and “I am no longer close to anyone”). Response options were 1 = “never”, 2 = “rarely”, 3 = “sometimes”, 4 = “most of the time”, 5 = “always”. The scale is considered reliable and valid [44]. The internal consistency in the sample waves was good (α = 0.86 (C5 & C6) to 0.88 (C7)). We calculated a total score ranging from 4 to 20. As there is no consensus cut-off value for loneliness, we applied a cut-off score of > 12 of the one-year prevalence, which was also used by previous authors [46]. This corresponds to an average rating that includes “most of the time” and “always”, making it a stricter criterion than the cut-off for the single item.

While the single loneliness measure was used across all 10 measurements, the UCLA Loneliness Scale was applied only for the last three COPSY waves. The correlation between the two loneliness measures ranged from *r* = 0.56 (C6) to 0.60 (C7) across measurement points.

### Sociodemographic variables

As sociodemographic information, we recorded age, gender, parental education, migration background, and single parenthood [see 56].

### Parental mental illness

Parents provided information answering a single item on any current psychiatric conditions that they themselves had been diagnosed with by a psychologist or physician.

### Mental health problems of children and adolescents

Mental health problems of children and adolescents were assessed with the internationally well-established Strengths and Difficulties Questionnaire (SDQ) [17]. It contains 20 items with response options from 0 = “not true”, 1 = “somewhat true”, to 2 = “certainly true”. Symptoms were rated over the last week. The total score of all items ranges from 0 to 40. Higher scores indicate more severe mental health problems. Cronbach’s α ranged from α = 0.82 (C1) to 0.86 (all other waves).

### Physical health complaints of children and adolescents

The 4-item physical health complaints (i.e., headache, stomachache, backache, and feeling dizzy) scale from the HBSC Symptom Checklist (HBSC-SCL) [18] was administered. Response options ranged from 1 = “rarely or never” to 5 = “about every day” during the past week. A mean item score ranging from 1 to 5 was calculated as the overall score, with higher values indicating more complaints. Cronbach’s α ranged from 0.69 (C3) to 0.73 (C7).

### Screen time

To measure screen time across digital devices (e.g., computer, smartphone, tablet, gaming console), we used a self-developed item asking about screen time for two distinct purposes: academic or educational activities (e.g., schoolwork) and personal use (e.g., leisure, entertainment). Respondents were asked to indicate the total hours spent daily with response options ranging from 1= “none” to 7 = “5 or more hours”.

### Personal resources

The Personal Resources Scale was used to capture individual capabilities such as self-efficacy, optimism, and a positive self-concept [3]. The five items (e.g., “I look to the future with optimism/confidence”) were provided with four response options (1 = “not true” to 4 = “exactly true”). The items were summed up and transformed into values between 0 and 100, with higher values indicating more personal resources. Cronbach’s α ranged from 0.81 to 0.86 across measurement points.

### Family cohesion

Family cohesion was assessed using the corresponding subscale of the Family Climate Scale (FSC) [45]. It covers feelings of connection and unity among members within a family. Each of the four items (e.g., “In our family everybody cares about each other’s worries”) was provided with four-point response options (1 = “not true” to 4 = “exactly true”). The items were summed up and transformed into values between 0 and 100, with higher values indicating higher family cohesion. Cronbach’s α varied from 0.86 to 0.89 across measurement points.

### Social support

Data on social support were collected using a short form with four items of the German translation of the Social Support Scale (SSS) [7]. The items (e.g., “How often has there been someone you can count on to listen to you when you need to talk”) were answered using five response options (1 = “never” to 5 = “always”). The items were summed up and transformed into values between 0 and 100, with higher values indicating higher family cohesion. Cronbach’s α ranged from 0.83 to 0.86 across measurement points.

Age, gender, loneliness (single item and UCLA Loneliness Scale), physical health complaints, screen time, personal resources, family cohesion, and social support were assessed by self-report. Parental education, migration background, single parenthood, parental mental illness, and mental health problems of children and adolescents were parent-reported.

### Data analysis

Descriptive analyses were carried out covering frequencies or means and standard deviations of all variables. To examine the course of loneliness, we took the weighted prevalence of the single item for each wave.

To test longitudinal changes in loneliness and to identify risk and resource factors associated with loneliness within (longitudinal) and between (cross-sectional) respondents, mixed model panel regression analyses were conducted. Since the UCLA Loneliness Scale was only used in COPSY waves 5–7 (2022–2024), we focused on these measurement points. Coefficients were estimated for exploring a potential effect of the time of the pandemic and other crises (dummy-variables for waves 6 and 7), time-constant risk factors (i.e., age, gender, migration background, parental education, single parenthood), time-varying risk- and resource factors (i.e., mental health problems and physical health complaints of children and adolescents, screen time, parental mental illness, personal resources, family cohesion, social support). The simultaneous inclusion of these predictors in the multivariate models allows for thoroughly controlling for confounding factors as well. In each model, a random intercept was included for every participant (to allow and represent individually differing scores). The random effects panel model was chosen because it allows the simultaneous inclusion of time-constant and time-varying covariates [53]. In addition, the association of each predictor alone was examined in a respective panel regression model with only the specific predictor specified as a fixed effect.

The required sample size was calculated using the software G-Power 3.1 and based on a cut-off for statistical significance of p (alpha) < 0.05 and a power of 80% for a small effect (f = 0.1) between two waves (within factor) and between two groups (between factor), and an interaction between two waves and two groups (within between interaction). This resulted in minimum sample sizes of *n* = 200 and *n* = 592, respectively. Missing data in single items was handled by exclusion of the particular cases from analyses incorporating the particular item. The panel regression analysis uses the full available data. The panel analyses were conducted using HLM8. For the remaining analyses, SPSS version 28 was used.

## Results

### Current feelings of loneliness (2022–2024)

In 2022, 10.2% of children and adolescents reported substantial loneliness over the past year, according to the UCLA Loneliness Scale. By 2023, this percentage had decreased to 8.2%. However, the downward trend did not continue in 2024, as 8.2% of children again reported feeling lonely (see Table 2).

**Table 2:** Frequency of loneliness by gender and age in 2022–2024 (one-year prevalence measured by UCLA loneliness Scale)

Year	Wave	*n*	All		Boys		Girls		11-13y		14-17y	
			**%**	(95% CI)	**%**	(95% CI)	**%**	(95% CI)	**%**	(95% CI)	**%**	(95% CI)
2022	C5	867	10.2	(8.2–12.2)	9.6	(6.8–12.4)	10.9	(7.9–13.8)	9.2	(6.2–12.2)	11.0	(8.2–13.7)
2023	C6	681	8.2	(6.1–10.2)	6.6	(4.0–9.3)	9.8	(6.6–13.0)	6.5	(3.7–9.3)	9.5	(6.5–12.4)
2024	C7	718	8.2	(6.2–10.2)	7.1	(4.5–9.7)	9.5	(6.4–12.6)	5.2	(2.7–7.6)	10.5	(7.6–13.5)

Children and adolescents reporting feelings of loneliness over the past year. Weighted data

**Fig. 1:**
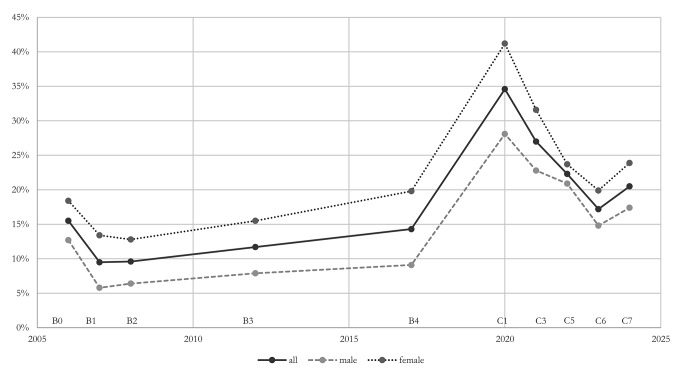
The longitudinal course of loneliness by gender. Loneliness was measured using a single-item question with a one-week timeframe. Data were weighted. In the BELLA studies, data collection spanned three years; for the figure, we used the final year to designate the measurement point

Looking at this three-year course, in 2022, 22.3% of all children reported some level of loneliness during the past week according to the single-item measure. While loneliness rates declined in 2023 to 17.2%, they rose again in 2024 to 20.5% (see Table 3, Fig. 1). Across the three years, the differences in loneliness between gender and age-groups were not statistically significant except for a higher proportion of girls experiencing loneliness in 2023 (*p* = 0.033).

**Table 3:** Frequency of loneliness by gender and age in 2003–2024 (one-week prevalence measured by single item)

Year	Wave	*n*	All		Boys		Girls		11-13y		14-17y	
			**%**	(95% CI)	**%**	(95% CI)	**%**	(95% CI)	**%**	(95% CI)	**%**	(95% CI)
2003–2006	B0	1734	15.5	(13.8–17.3)	12.7	(10.4–15.0)	18.4	(15.7–21.2)	9.0	(6.8–11.3)	19.7	(17.2–22.2)
2004–2007	B1	1497	9.5	(8.1–11.0)	5.8	(4.2–7.4)	13.4	(11.0–15.8)	7.4	(5.2–9.6)	10.2	(8.1–12.3)
2005–2008	B2	1414	9.6	(8.2–11.0)	6.4	(4.7–8.0)	12.8	(10.5–15.1)	7.6	(5.3–9.8)	9.1	(7.0–11.1)
2009–2012	B3	1282	11.7	(9.9–13.5)	7.9	(5.7–10.0)	15.5	(12.6–18.4)	7.8	(4.9–10.7)	13.5	(11.2–15.8)
2014–2017	B4	1660	14.3	(14.2–14.3)	9.1	(9.1–9.2)	19.8	(19.8–19.9)	8.6	(8.5–8.6)	17.3	(17.2–17.3)
2020	C1	1040	34.6	(31.7–37.5)	28.1	(24.3–32.0)	41.2	(36.9–45.5)	35.9	(31.3–40.4)	33.7	(29.9–37.5)
2021	C3	1043	27.0	(24.5–29.6)	22.8	(19.5–26.1)	31.6	(27.7–35.4)	23.1	(19.1–27.1)	31.0	(27.2–34.7)
2022	C5	833	22.3	(19.9–24.7)	20.9	(17.6–24.3)	23.7	(20.2–27.2)	21.2	(17.0–25.4)	23.9	(20.1–27.6)
2023	C6	708	17.2	(15.1–19.3)	14.8	(12.0–17.5)	19.9	(16.6–23.2)	16.9	(13.0–20.8)	15.9	(12.6–19.3)
2024	C7	640	20.5	(18.1–23.0)	17.4	(14.2–20.6)	23.9	(20.2–27.7)	19.4	(15.0–23.9)	22.9	(18.8–27.0)

Children and adolescents reporting to feel “sometimes”, “almost always”, or “always” lonely over the last week. Weighted data

**Table 4:** Risk and resource factors of loneliness in children and adolescents

	Bivariate model			Multivariate model		
	B	95% CI		B	95% CI	
		LL	UL		LL	UL
Intercept	1.681^a^	1.641 ^a^	1.720 ^a^	1.001	0.864	1.139
Female Gender	0.055	-0.012	0.122	0.077	0.018	0.136
Age (years)	0.006	-0.004	0.016	0.006	-0.007	0.020
Migration background	0.024	-0.068	0.116	-0.024	-0.097	0.049
Low parental education	-0.142	-0.246	-0.038	-0.123	-0.197	-0.049
Single parenthood	-0.063	-0.153	0.027	0.026	-0.052	0.104
Screen time school (hours)	-0.014	-0.030	0.002	-0.001	-0.018	0.016
Screen time private (hours)	0.053	0.033	0.073	0.006	-0.016	0.027
Parental mental illness	0.495	0.387	0.603	-0.019	-0.137	0.099
Mental health problems	0.067	0.063	0.071	0.045	0.038	0.052
Physical health complaints	0.394	0.331	0.456	0.052	0.127	0.127
Personal resources	-0.017	-0.019	-0.015	-0.006	-0.009	-0.004
Family cohesion	-0.014	-0.016	-0.012	0.000	-0.002	0.002
Social support	-0.017	-0.019	-0.015	-0.008	-0.010	-0.005
COPSY Wave 6 (2023)	-0.068	-0.113	-0.023	-0.033	-0.082	0.017
COPSY Wave 7 (2024)	-0.101	-0.150	-0.052	-0.040	-0.095	0.014
Model fit (R2)				0.24 person level	0.57 person and time level	

Results of mixed model panel regression analyses. The parameter estimates indicate how the predictors are associated with the outcome UCLA loneliness scored 1–5 instead of 4–20 to enable better interpretation of effects. Unweighted data. ^a^ issued from base model with random intercept only. Parental education low vs. medium/high. Data form COPSY wave 5 (2022), wave 6 (2023), and wave 7 (2024)

It is also noteworthy that about half of the young people reported „never“ feeling lonely (see Supplementary Table S1). In 2024, (51.4%) of the young people reported „never“ feeling lonely in the past week, and an additional 27.2% said they „rarely“ felt lonely. However, 16.6% experienced loneliness „sometimes“, and a smaller but notable group (3.7%) felt lonely „often“, and 1.1% felt „always“ lonely (see Supplementary Table S1).

### The course of loneliness over the past 20 years (2003–2024)

Loneliness prevalence among young people in Germany has shown significant fluctuations over the past 20 years (see Fig. 1 and Supplement Figure S1). At our first measurement (single item) taken between 2003 and 2006, the prevalence of loneliness was 15.5%. By the one-year follow-up, it had dropped to 9.5% (*p* < 0.001; V = 0.09) and remained stable at 9.6% during the two-year follow-up (*p* = 0.950; V = 0.00). However, six years after the baseline in 2009–2012, loneliness increased again (but not statistically significant), reaching 11.7% (*p* = 0.069; V = 0.03). By the eleven-year follow-up in 2014–2017, the rate had risen further to 14.3%, again, the increase was not statistically significant (*p* = 0.079; V = 0.04) (see Table 3). But the increase between 2005 and 2008 (B2) and 2014–2017 (B4) was significant (*p* < 0.001; V = 0.07).

At the start of the COVID-19 pandemic in 2020, the prevalence of loneliness was significantly higher. During this time, 34.6% of young people in Germany reported feeling lonely at least sometimes during the previous week. This figure declined in subsequent years, dropping to 27.0% in 2021, 22.3% in 2022, and 17.2% in 2023. However, by 2024, loneliness prevalence rose again slightly to 20.5%. Overall, loneliness has declined since the start of the pandemic, but remains above pre-2020 levels (see Table 3).

Throughout the entire time between 2003 and 2024, girls consistently reported higher levels of loneliness than boys, only in COPSY wave 5 in 2022, where the difference was statistically not significant (*p* = 0.191– *p* < 0.001; V = 0.04–0.16). Pre-pandemic data showed that girls were twice as likely to experience loneliness as boys. At the start of the pandemic, a high number of 41.2% of girls felt lonely compared to 28.2% of boys. After the pandemic, the rates of loneliness between girls and boys became somewhat more similar.

From 2003 until the pandemic, older adolescents (14–17 years) experienced more loneliness than younger ones (11–13 years), yet these differences were statistically significant only at the BELLA baseline (B0), the six-year (B3), and the eleven-year follow-up (B4) (*p* = 0.353– *p* < 0.001; V = 0.03–0.14). However, with the onset of the pandemic in 2020, younger participants reported higher rates of loneliness than older ones yet this difference (35.8% vs. 33.7%) was statistically not significant (*p* = 0.475; V = 0.02). In the subsequent waves during the pandemic (in C3, C5), older adolescents reported again more loneliness than younger ones. Yet, these differences were statistically significant only in COPSY wave 3 in 2021 (*p* = 0.680– *p* = 0.006; V = 0.01–0.09) (see Table 3 and Supplement Figure S1).

### Risk and resource factors of loneliness in children and adolescents

Bivariate regression model analyses showed that risk factors such as having mental health problems and physical health complaints, parental mental illness, and longer screen time for private use were linked to higher loneliness in children and adolescents. Conversely, low parental education, higher personal resources, strong family cohesion, and more social support were associated with lower loneliness in the bivariate models (not controlled for all other predictors). Compared to COPSY wave 5 (C5, i.e., first wave after the pandemic), wave 6 (C6, 2023) and wave 7 (C7, 2024) were linked to a lower chance of feeling lonely.

In the multivariate panel regression analysis on loneliness, we found that female gender, having mental health problems, and physical health complaints were linked to higher loneliness scores in children and adolescents. Again, low parental education, higher personal resources, and strong family cohesion were associated with lower loneliness scores.

The panel regression model explained 24% of the total variance in loneliness between respondents and 57% of the total variance within (over time) and between respondents. For descriptive data on risk and protective factors, see Table S2 in the supplement.

## Discussion

Loneliness has increasingly been recognized as a critical public health concern, as highlighted by global health authorities, such as the World Health Organization and the U.S. Surgeon General [15, 22]. Our study is the first comprehensive longitudinal, population-based study in Germany, leveraging large, methodologically comparable datasets to reveal the extent of loneliness among children and adolescents over the past two decades.

Our data from 2003 to 2024 shows notable fluctuations of loneliness, with a sharp increase during the COVID-19 pandemic. At the baseline measurement (2003–2006), 15.5% of young people reported feeling lonely sometimes or more often, with a significant decrease by 6% at the one- and two-year follow-ups. One possible explanation for this drop is that it may have been easier to disclose loneliness in writing (at baseline) than voicing it during the phone interview (at follow-ups), as individuals tend to give socially desirable responses [26]. Subsequently, our measure shows a steady increase in loneliness. While the changes between individual measurement points were not statistically significant, the overall increase from 2005 to 2008 to 2014–2017 reached statistical significance. Possible explanations for the increase in loneliness even before the COVID-19 pandemic may include an increase in social media consumption [49, 50], a decrease in face-to-face contacts with the peer group [49], an increase in mental illnesses [5], and a fragmentation of social networks as well as greater mobility [5]. Other studies focusing specifically on loneliness at school report an even more pronounced increase between 2012 and 2018 [50], suggesting that social exclusion and feelings of loneliness within the school environment may be major contributors to rising levels of loneliness and decreased well-being among young people.

In 2020, loneliness spiked dramatically in our study to 34.6%, most likely due to pandemic-related social restrictions, isolation, and disruptions in education and social life. Nevertheless, it should also be noted that there is no longitudinal comparison between 2017 and 2020, so it could also be that the COPSY 2020 sample was generally lonelier than the pre-pandemic sample in 2017. However, an increase in loneliness during the pandemic is in alignment with other cross-sectional and longitudinal studies [12, 47]. It is worth noting, however, that some studies did not report a significant additional rise in loneliness during COVID-19 [52]. One possible explanation for these divergent findings is the difference in the severity of the pandemic and the protective measures implemented in each country.

In the subsequent years, our data showed a steady decline in loneliness (27.0% in 2021, 22.3% in 2022, and 17.2% in 2023), which could be interpreted as a partial recovery from pandemic-related loneliness. However, in the most recent measurement in 2024, the percentage of young people feeling lonely (20.5%) remains substantially higher, by 6% points, compared to pre-pandemic assessments. However, the recovery observed in our study is less pronounced than observed in older age groups [51], which may suggest that younger individuals continue to struggle with the long-term consequences of the pandemic. This is particularly plausible given that young people experienced some of the strictest restrictions during the pandemic in Germany. Additionally, other factors could be contributing to current feelings of loneliness in young people, including sustained high levels of media consumption, ongoing reductions in face-to-face social interactions, and widespread concerns about global issues such as wars, inflation, and climate change [23].

The observed increase in reported loneliness over time might, in part, be attributable to the Prevalence Inflation Hypothesis [13], which suggests that growing awareness of mental health may lead to inflated reporting. Increased sensitivity to emotional states could cause young people to interpret normal fluctuations as loneliness. Media attention during the pandemic and reduced stigma may have further encouraged more open acknowledgment of these experiences.

In addition to the single-item measure assessing loneliness over the past week, we employed the UCLA Loneliness Scale to assess experiences of loneliness over the past twelve months at the three most recent survey waves (2022–2024). About one in ten young people in Germany reported substantial (“most of the time” and “always”) loneliness over the past year. We further used data from these three assessments to examine risk and protective factors associated with loneliness. Consistent with our expectation and other research, female gender was a risk factor for loneliness in our multivariate model [27, 47]. Possible explanations include differences in socialization, with girls being more likely to value social relationships and express their emotions, making them more aware of loneliness [14]. Additionally, girls are more vulnerable to internalizing mental health issues, which are linked to more loneliness [12, 27].

Contrary to our expectations and previous research [9], older age did not emerge as a risk factor for loneliness when controlling for all other predictors in our multivariate regression analysis. But bivariate analyses of the single item measure indicated higher symptoms for older than younger children for some measurement points (B0, B3, B4, and C3). These mixed findings suggest a complex relationship between age and loneliness in young people. A more detailed investigation of this relationship is beyond the scope of the current paper and warrants further research.

In addition, and surprisingly, low parental education was associated with lower loneliness. This is not in line with other research showing that low family affluence is associated with higher loneliness [47], a well-established finding in research of mental health problems in young people [43]. In the context of the COVID-19 pandemic, interestingly, some studies showed no longer differences in loneliness depending on education and income [10]. This may be because, during the coronavirus crisis, higher socio-economic status no longer provided greater opportunities for social participation, as protective measures impacted everyone equally [10].

Loneliness among adolescents was associated with poorer self-reported physical health complaints, like headaches and stomachaches, conforming to previous research [9, 27]. We also found a significant association between loneliness and mental health problems. This is in line with previous research [12, 25]. Loneliness can both predict and be a consequence of poor mental health. On one hand, chronic loneliness has been linked to increased risks of mental health disorders such as depression and anxiety [19, 25, 34]. On the other hand, existing mental health problems like depression or anxiety can lead to loneliness [29], creating a vicious cycle with each condition reinforcing the other. Future research needs to investigate the interplay of loneliness and mental health problems in young individuals.

Personal and social resources were associated with lower loneliness in our multivariate model, aligning with both theoretical expectations and our previous research [24, 56]. Consistent with international studies, young people who have social and personal resources are at lower risk of experiencing loneliness [21, 29, 55]. This finding highlights a key leverage point for prevention and intervention.

Turning to the practical implications of our study, research suggests that youth loneliness can be reduced through targeted interventions [8], highlighting the role of schools, communities, healthcare providers, and policymakers in addressing this issue. However, a clear definition of chronic loneliness and a better understanding of which evidence-based intervention strategies are most effective in different groups and contexts are needed. Connecting youth to out-of-school programs, such as sports, arts, church groups, and volunteering, can foster meaningful relationships and a sense of belonging. One promising approach is social prescribing, where healthcare professionals refer individuals to community-based activities and support networks [2, 30]. Furthermore, schools play a crucial role in reducing loneliness by fostering supportive environments, raising awareness, and equipping students with social-emotional skills. Early interventions, such as school-based screening programs, can help identify at-risk youth and provide timely support, preventing loneliness from becoming chronic.

Our study has several strengths and limitations. The strengths include large population-based samples, trend data on loneliness over 20 years, the administration of diverse established questionnaires, which are comparable across the two large studies involved (BELLA/COPSY), and the application of advanced methodology (panel regression analyses) on a longitudinal data set. Baseline participation rates were consistent with other child health surveys [16]. Our study is also subject to several limitations. A limitation of our study is the use of two separate longitudinal datasets, which prevented us from tracking the same individuals and conducting longitudinal analyses across the entire study period. Additionally, loneliness was measured using the UCLA Loneliness Scale only from 2022 onward, while for the entire time span, only a single-item measure of loneliness was available. Furthermore, in the BELLA study, data collection took place over a three-year period rather than annually, resulting in the absence of yearly measurements. Our findings may also be affected by response biases, such as social desirability bias or nonresponse bias. Furthermore, our results may not be generalizable beyond Germany. Like all studies, ours focused on specific risk and protective factors, potentially overlooking other relevant influences.

## Conclusion

While loneliness is a common experience among children and adolescents, it becomes a critical concern when it persists and negatively affects physical and mental health. Our study results highlight the urgent need for targeted screening and intervention programs, as there remains a significant gap in well-structured, evidence-based approaches. Addressing loneliness requires a multi-level strategy, integrating both school-based and community-level initiatives. Schools provide an ideal setting to reach large groups of young people and foster essential emotional and social skills. Additionally, local extracurricular activities - such as sports, music, arts, and volunteering in community organizations - can promote meaningful social connections and a sense of belonging.

Loneliness is increasingly recognized as a critical public health concern, as highlighted by global health authorities and our study findings. Addressing loneliness is not just essential for enhancing immediate well-being, but also for preventing future psychological distress and improving overall public mental and physical health outcomes. A concerted effort to give young people a sense of community and belonging will foster their resilience and contribute to a healthier future.

## Electronic supplementary material

Below is the link to the electronic supplementary material.

Supplementary Material 1

## Data Availability

The data that support the findings of this study are available from the corresponding author upon reasonable request.
