# Supplementary material for: Two decades of loneliness among children and adolescents: longitudinal trends, risks and resources – Results from the German BELLA and COPSY studies
Source: Eur Child Adolesc Psychiatry. 2025 Jun 9;34(11):3629–41. doi: 10.1007/s00787-025-02779-6 (PMC12647284; doi:10.1007/s00787-025-02779-6)
Supplement: Supplementary file 1 — Supplementary Material 1 [file 787_2025_2779_MOESM1_ESM.docx]

Online supplement for Zoellner et al. “Two decades of loneliness among children and adolescents: Longitudinal trends, risks and resources – Results from the German BELLA and COPSY studies”

Corresponding authors: Prof. Ulrike Ravens-Sieberer ([ravens-sieberer@uke.de](mailto:ravens-sieberer@uke.de)) and Dr. Anne Kaman (a.kaman@uke.de)

# SUPPLEMENTARY TABLE S1. Frequency of loneliness by response category in 2022-2024

|  | **2022 (C5)** | | **2023 (C6)** | | **2024 (C7)** | |
| --- | --- | --- | --- | --- | --- | --- |
|  | n | % | n | % | n | % |
| never (1) | 401 | 46.3 | 346 | 50.8 | 369 | 51.4 |
| rarely (2) | 269 | 31.0 | 215 | 31.5 | 195 | 27.2 |
| sometimes (3) | 145 | 16.7 | 95 | 13.9 | 119 | 16.6 |
| often (4) | 46 | 5.3 | 19 | 2.8 | 26 | 3.7 |
| always (5) | 6 | 0.7 | 6 | 0.9 | 8 | 1.1 |

Loneliness measured by a single item. Time frame: Past week. Weighted data.

# SUPPLEMENTARY TABLE S2. Descriptive data of loneliness, risk and resource factors

|  | **Range of raw scores** | **2022 (C5)**  **n = 833** | **2023 (C6)**  **n = 708** | **2024 (C7)**  **n = 640** |
| --- | --- | --- | --- | --- |
|  |  | M (SD) | M (SD) | M (SD) |
| **Loneliness** |  |  |  |  |
| UCLA Loneliness Scale | 0.25-5 | 1.71 (0.76) | 1.62 (0.74) | 1.58 (0.71) |
| **Risk factors** |  |  |  |  |
| Parental mental illness | 0-1 | 0.06 (0.24) | 0.09 (0.29) | 0.84 (0.28) |
| Mental health problems | 0-40 | 8.92 (6.25) | 9.02 (6.30) | 8.56 (6.19) |
| Physical health complaints | 1-5 | 1.51 (0.58) | 1.43 (0.55) | 1.62 (0.67) |
| Screen time - school | 1-7 | 3.32 (1.58) | 3.33 (1.55) | 3.50 (1.81) |
| Screen time - private | 1-7 | 5.0 (1.43) | 4.90 (1.47) | 4.97 (1.55) |
| **Resource factors** |  |  |  |  |
| Personal resources | 0-100 | 68.47 (18.82) | 70.13 (17.57) | 70.32 (18.51) |
| Family cohesion | 0-100 | 75.34 (18.50) | 76.24 (18.81) | 76.63 (18.93) |
| Social support | 0-100 | 79.20 (17.09) | 79.18 (16.76) | 80.06 (17.10) |

M= mean; SD=standard deviation. Weighted data. Data from COPSY wave 5 (2022), 6 (2023), and 7 (2024).

# SUPPLEMENTARY FIGURE S1. The longitudinal course of loneliness by age


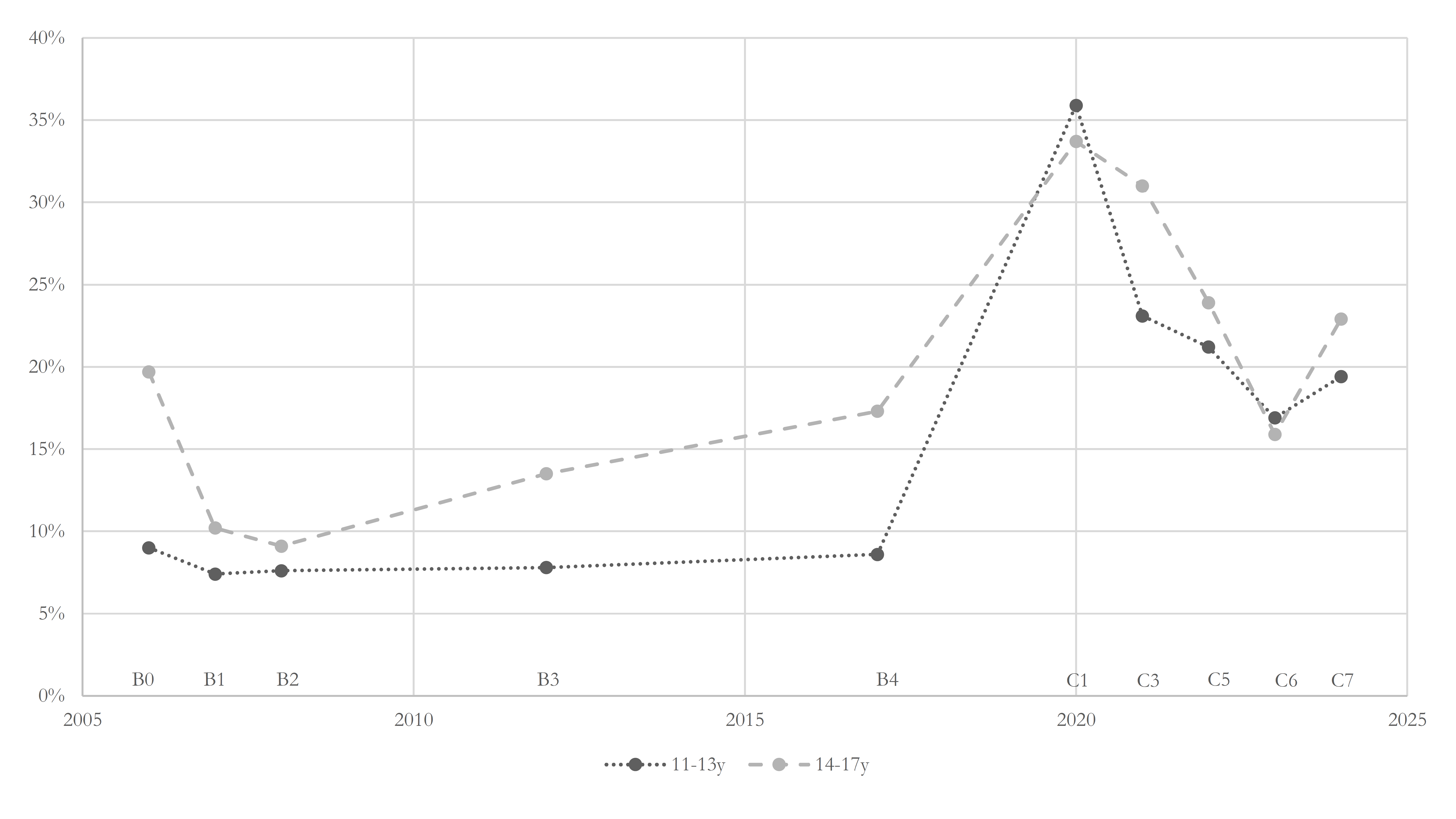


Loneliness was measured using a single-item question with a one-week timeframe. Data were weighted. In the BELLA studies, data collection spanned three years; for the figure, we used the final year of this time span to designate the measurement point.
